# Cost‐effectiveness analysis of osimertinib plus chemotherapy for patients with EGFR‐mutated advanced non‐small cell lung cancer

**DOI:** 10.1002/cam4.70083

**Published:** 2024-08-29

**Authors:** Wentao Tian, Lishui Niu, Rongrong Zhou, Ziqi Wang, Jiaoyang Ning, Ruoyu Lu, Yin Shi, Zhaohua Tan

**Affiliations:** ^1^ Department of Oncology Xiangya Hospital, Central South University Changsha Hunan China; ^2^ Xiangya Lung Cancer Center Xiangya Hospital, Central South University Changsha China; ^3^ National Clinical Research Center for Geriatric Disorders Xiangya Hospital, Central South University Changsha China; ^4^ Department of Pharmacy Xiangya Hospital, Central South University Changsha Hunan China

**Keywords:** chemotherapy, cost‐effectiveness, EGFR, non‐small cell lung cancer, osimertinib

## Abstract

**Introduction:**

First‐line osimertinib plus chemotherapy significantly prolonged progression‐free survival of patients with EGFR‐mutated advanced non‐small cell lung cancer (NSCLC) compared to osimertinib, according to the FLAURA2 trial.

**Methods:**

We established a Markov model to compare the cost‐effectiveness of osimertinib plus chemotherapy with that of osimertinib alone. Clinical data were obtained from the FLAURA and FLAURA2 trials, and additional data were extracted from online resources and publications. Sensitivity analyses were conducted to evaluate the robustness of the findings. We used A willingness‐to‐pay threshold of $150,000 per quality‐adjusted life‐years (QALYs) gained. The main outcomes were QALYs, overall costs, incremental cost‐effectiveness ratio (ICER), incremental net monetary benefit, and incremental net health benefit. Subgroup analyses were conducted according to patients' mutation type and central nervous system (CNS) metastatic status.

**Results:**

In a 20‐year time horizon, the ICER of osimertinib plus chemotherapy versus osimertinib alone was $223,727.1 per QALY gained. The sensitivity analyses identified the cost of osimertinib and the hazard ratio for overall survival as the top 2 influential factors and a 1.9% probability of osimertinib plus chemotherapy to be cost‐effective. The subgroup analyses revealed ICERs of $132,614.1, $224,449.8, $201,464.1, and $130,159.7 per QALY gained for L858R mutations, exon 19 deletions, CNS metastases, and no CNS metastases subgroups, respectively.

**Conclusions:**

From the perspective of the United States health care system, osimertinib plus chemotherapy is not cost‐effective compared to osimertinib alone for treatment‐naïve patients with EGFR‐mutated advanced NSCLC, but more favorable cost‐effectiveness occurs in patients with L858R mutations and patients without baseline CNS metastases.

## 1 INTRODUCTION

Lung cancer ranks as the primary cause of cancer‐related mortality and the second most prevalent malignant neoplasm.^1^ Non‐small cell lung cancer (NSCLC) represents the predominant histopathological subtype, encompassing approximately 85%–90% of all lung cancer cases.^2^ The identification of epidermal growth factor receptor (EGFR) sensitive mutations in 2004 has emerged as a pivotal factor in the progression of NSCLC and is closely linked to the responsiveness to EGFR‐tyrosine kinase inhibitors (EGFR‐TKIs).^3^, ^4^ Moreover, previous investigations have demonstrated substantial inhibitory effects of EGFR‐TKIs on NSCLC harboring EGFR‐activating mutations.^4^

According to certain guidelines, EGFR‐TKIs are currently recommended as the standard initial treatment for patients with EGFR mutation‐positive advanced NSCLC.^5^ Osimertinib, a third‐generation TKI, has been widely used in advanced NSCLC with mutations due to its superior therapeutic efficacy in inhibiting both EGFR‐TKI‐sensitizing and EGFR p.Thr790Met (T790M) resistance mutations.^6^ The phase III RCT, FLAURA, demonstrated that osimertinib achieved higher progression‐free survival (PFS) (18.9 vs. 10.2 months, hazard ratio [HR] = 0.46, 95% confidence interval [CI]: 0.37–0.57, *p* < 0.001) and overall survival (OS) (38.6 vs. 31.8 months, HR = 0.80, 95.05% CI: 0.64–1.00, *p* = 0.046) rates compared to first‐generation EGFR‐TKIs.^7^ Consequently, based on the findings of FLAURA, osimertinib has been considered the preferred treatment approach for NSCLC.^8^

However, the use of EGFR‐TKIs as a standalone treatment still resulted in disease progression in most patients. As a potential solution, it has been suggested that incorporating a platinum‐based agent and pemetrexed alongside EGFR‐TKIs may potentially prolong survival compared to using EGFR‐TKIs alone.^9^ Several phase 2 and 3 trials have compared the efficacy of combining first‐generation EGFR‐TKIs with chemotherapy versus using first‐generation EGFR‐TKIs alone, and these trials have consistently demonstrated that the combined regimen yields superior efficacy outcomes, thus supporting the aforementioned hypothesis.^10^, ^11^, ^12^ Consequently, the investigation of combined third‐generation EGFR‐TKIs and chemotherapy is currently a topic of great research interest.

The FLAURA2 study evaluated the efficacy of osimertinib combined with chemotherapy in patients with advanced mutated NSCLC. Results from this phase 3 trial demonstrated a significantly longer progression‐free survival (PFS) in the osimertinib plus chemotherapy group compared to the osimertinib alone group (29.4 vs. 19.9 months, HR, 0.62; 95% CI, 0.48–0.80).^9^ Although osimertinib combined with chemotherapy could provide benefits for the patients, the cost of osimertinib plus chemotherapy is higher compared to the cost of osimertinib alone. In this study, we conducted a cost‐effectiveness analysis to assess the economic viability of utilizing osimertinib in combination with chemotherapy as the initial treatment for untreated advanced non‐small cell lung cancer (NSCLC) with EGFR mutations in the United States setting based on the results from the FLAURA2 trial and the FLAURA trial.

## 2 MATERIALS AND METHODS

This study adhered to the Consolidated Health Economic Evaluation Reporting Standards 2022 (CHEERS 2022). All statistical analyses were carried out using the R software (version 4.2.1). We adopted the “heemod” package for Markov model‐based cost‐effectiveness analyses,^13^ the “IPDfromKM” package for reconstruction of survival data,^14^ and the “flexsurv” package for curve fitting.^15^

### 2.1 Model structure

**FIGURE 1:**
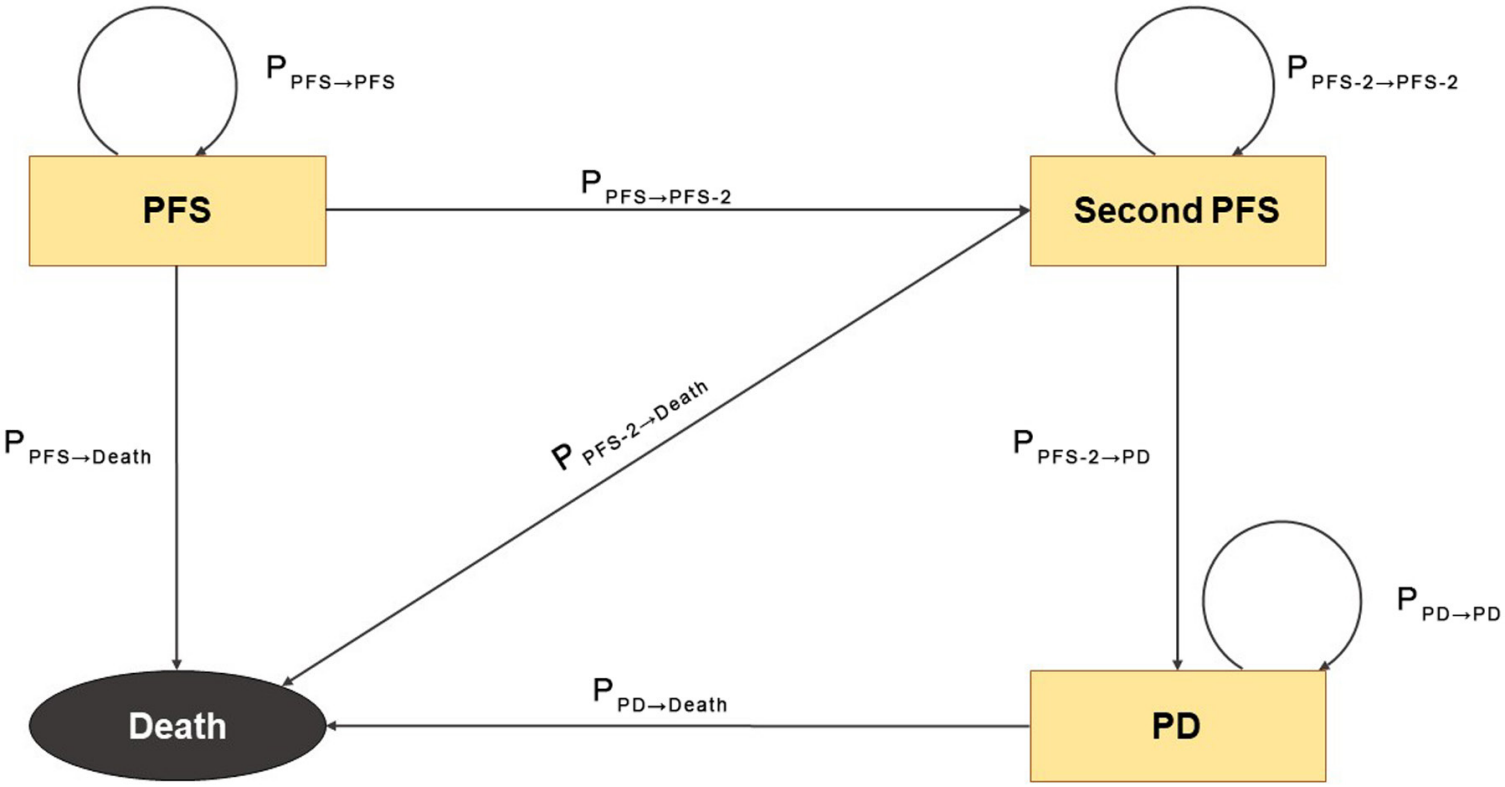
Model structure. PD, progressive disease; PFS, progression‐free survival.

A Markov model was constructed to assess the health outcomes and cost‐effectiveness of initial treatment strategies for advanced mutation‐NSCLC.^13^ The model operated on 21‐day cycles, as depicted in Figure 1. The analysis was conducted from the perspective of the health care system in the United States. The FLAURA2 trial, which encompassed 557 untreated adult patients with locally advanced or metastatic NSCLC and EGFR exon 19 deletion (Ex19del) or p.Leu858Arg (L858R) mutation, provided the basis for the study. The patients were allocated randomly in a 1:1 ratio to receive either osimertinib plus chemotherapy or osimertinib monotherapy. In our model, we established a 20‐year time horizon, as we assumed that the therapeutic effects of both treatments would be equivalent after 20 years from the commencement of therapy. This model incorporates four states, namely PFS, second PFS (PFS‐2), progressive disease (PD), and death, with death serving as the absorbing state. The PFS state referred to the state where patients receive first‐line treatments without disease progression, while the PFS‐2 referred to the state where patients had their earliest progression following first‐line treatment and received second‐line therapies. Patients in the PD state had further progressions following second‐line treatments. All patients were initially enrolled in the PFS health state and subsequently treated with either first‐line osimertinib monotherapy or a combination of osimertinib and chemotherapy until disease progression or intolerable toxicity. Patients who experienced progression during first‐line treatment were transitioned to the PFS‐2 state, where they received second‐line anticancer therapies. Patients in the PFS‐2 state were also eligible to transition to the PD health state after the second progression. Ultimately, patients in any of the states could directly progress to the Death state.

The transition probabilities from the other states to the progression or death state were estimated using time‐dependent data from the trial's PFS, PFS‐2, and OS, as well as the automatically obtained natural mortality rate per cycle (*P*_Natural_) from the “heemod” package.^13^ The non‐zero transition probabilities from state A to state B, *P*_A→B_, at the *t* ^th^ Markov cycle were displayed in Figure 1 and defined as follows:

$$P_{\mathrm{PFS}\to \mathrm{PFS}-2}=\frac{P\left(t\right)-P\left(t+1\right)}{P\left(t\right)}-P_{\text{Natural}}$$

$$P_{\mathrm{PFS}-2\to \mathrm{PD}}=\frac{P_{2}\left(t\right)-P_{2}\left(t+1\right)}{P_{2}\left(t\right)}-P_{\text{Natural}}$$

$$P_{\mathrm{PD}\to \text{Death}}=\frac{S\left(t\right)-S\left(t+1\right)}{S\left(t\right)-P_{2}\;\left(t\right)}-P_{\text{Natural}}$$

*P*(*t*), *P*_2_(*t*), and *S*(*t*), estimated by the fitted curves, were the probabilities of PFS, second PFS, and OS at the beginning of the *t* ^th^ Markov cycle. *P*_PFS→Death_ and *P*_PFS‐2→Death_ were set to *P*_Natural_. *P*_PFS→PFS_, *P*_PFS‐2→PFS‐2_, *P*_PD→PD_, and *P*_Death→Death_ were defined as 1 − *P*_PFS→Death_ − *P*_PFS‐PFS‐2_, 1 − *P*_PFS_ − _2→Death_ − *P*_PFS‐2→PD_, 1 − *P*_PD→Death_, and 1, respectively. Probabilities were reasonably adjusted to 0 or 1 when they fell out of the interval between 0 and 1.

The primary outcomes of this study included the quality‐adjusted life‐years (QALYs), overall costs, incremental cost‐effectiveness ratios (ICERs), incremental net health benefit (INHB) and incremental net monetary benefit (INMB) at a willingness‐to‐pay (WTP) threshold of $150,000 per QALY gained. To account for the time value of money, a discount rate of 3% per year was applied to both health outcomes and costs.

### 2.2 Clinical data

The PFS and second PFS Kaplan–Meier (K–M) curve data points from the FLAURA2 trial were obtained using the GetData Graph Digitizer (version 2.26). As the FLAURA2 trial did not provide a mature K–M curve for OS, the survival data points for osimertinib monotherapy were extracted from the FLAURA OS curve.^7^ The characteristics of the two osimertinib groups, including age, sex, race, WHO performance‐status score, EGFR mutation status, overall disease classification, and histologic type in the FLAURA and the FLAURA2 trial were compared to ensure the reasonability of the OS data substitution (Table S1). The OS curve for osimertinib plus chemotherapy was estimated by applying the hazard ratio between the osimertinib mono‐therapy group and the osimertinib plus chemotherapy group in the FLAURA2 trial.^14^ The individual patient data were reconstructed using the “IPDfromKM” package,^16^ and K–M curves were generated to ensure accuracy (Figure S1). Long‐term PFS, second PFS, and OS curves were modeled using various distribution functions, including exponential, Weibull, log‐logistic, log‐normal, generalized gamma, gamma, and Gompertz, with the “flexsurv” package (Figure S2). Subsequently, the best‐fit distribution models were selected based on a combination of Akaike Information Criterion (AIC) and Bayesian Information Criterion (BIC) values, as well as visual inspections (Table S2). In some cases, the fitting curves with the lowest AIC/BIC were not chosen due to tailing or PFS being higher than OS. Additional clinical data, including the rates of drug discontinuation, occurrences of severe adverse events (SAEs), follow‐up time, response duration, the count of patients administered osimertinib in combination with platinum‐pemetrexed or osimertinib monotherapy, and the number of patients who underwent subsequent treatments, were also collected.

### 2.3 Cost and utility data

This study primarily examines the direct medical costs associated with patients' therapeutic phase in the United States. These costs encompass various components such as first‐line and subsequent drug expenses, EGFR mutation testing costs, intravenous infusion expenses, trimonthly imaging costs, routine follow‐up expenses, end‐of‐life care costs (spanning 6 months), best supportive care (BSC) expenses, and management of AEs as outlined in Table 1. Following the design of the FLAURA2 trial, patients assigned to the combination arm were administered osimertinib at a dosage of 80 mg once daily, along with intravenous pemetrexed at a dosage of 500 mg per square meter of body surface area (BSA). Additionally, they received either cisplatin at a dosage of 75 mg per square meter or carboplatin at a dosage determined by pharmacologically guided means, specifically an area under the concentration‐time curve of 5 mg/mL/min. These medications were administered intravenously on Day 1 of each 21‐day cycle for a total of four cycles. Subsequently, the patients underwent maintenance therapy consisting of osimertinib at a dosage of 80 mg once daily, in combination with pemetrexed at a dosage of 500 mg per square meter, administered every 21 days. The treatment regimen described above served as the experimental group, while the control group received a different intervention. The control arm exclusively received osimertinib at a daily dosage of 80 mg until the manifestation of PD. Based on the data published in the FLAURA2 study, patients primarily underwent treatment with chemotherapy, EGFR‐TKIs, VEGF inhibitors, PD‐1 inhibitors, or PD‐L1 inhibitors as subsequent therapies for their cancer. To accurately represent the clinical practice, it was assumed that patients would receive BSC before their death. Moreover, it was assumed that all patients would undergo genetic testing upon initial diagnosis, followed by monthly physician visits and imaging examinations trimonthly as part of the routine follow‐up protocol. To determine the appropriate dosage of these agents, a standard 65‐year‐old patient with a weight of 70 kg, a BSA of 1.86 m^2^, and a creatinine clearance rate (Ccr) of 70 mL/min was used as a reference.^17^, ^18^, ^19^ The costs associated with these treatments were obtained from the Centers for Medicare & Medicaid Services and various published articles, accounting for inflation to 2023.^25^, ^26^ The utility‐scale ranged from 0 (representing death) to 1 (indicating perfect health), with distinct utilities assigned to specific health states. We consulted previously published articles to acquire utility values linked to survival and health states, as well as disutility values associated with SAEs (Table 1).^20^, ^23^, ^27^

**TABLE 1:** Parameters input to the model.

Parameters	Baseline Value	Range		Distribution	Source
		Minimum	Maximum		
Clinical					
Body weight	70	52.5	87.5	Normal	Goulart B, et al.^17^
BSA	1.86	1.40	2.33	Normal	Kohn CG, et al.^18^
Ccr	70	52.5	87.5	Normal	Liu Q, et al.^19^
Discount rate	0.03	ND	ND	Uniform	Kohn CG, et al.^18^
HR for OS	0.75	0.57	0.97	Lognormal	Planchard D, et al.^9^
Osimertinib discontinuation rate in the OC group	0.4420	0.3978	0.4862	Beta	Planchard D, et al.^9^
Carboplatin/cisplatin discontinuation rate in the OC group	0.2319	0.2087	0.2551	Beta	Planchard D, et al.^9^
Pemetrexed discontinuation rate in the OC group	0.7536	0.6783	0.8290	Beta	Planchard D, et al.^9^
Osimertinib discontinuation rate in the O group	0.5527	0.4975	0.6080	Beta	Planchard D, et al.^9^
Subsequent treatment discontinuation probability per cycle	0.0300	0.0270	0.0330	Beta	Estimated
Transition probabilities (at the t^th^ Markov cycle)^a^					
*P*_PFS→PFS‐2_	(*P*(t) − *P*(t + 1))/*P*(t) – *P*_Natural_	NA	NA	NA	Curve fitting
*P*_PFS‐2→PD_	(*P*_2_(t) − *P*_2_(t + 1))/*P*_2_(t) − *P*_Natural_	NA	NA	NA	Curve fitting
*P*_PD→Death_	(S(t) − S(t + 1))/(S(t) − *P*_2_(t + 1)) − *P*_Natural_	NA	NA	NA	Curve fitting
*P*_PFS→Death_	*P*_Natural_	NA	NA	NA	Curve fitting
*P*_PFS‐2→Death_	*P*_Natural_	NA	NA	NA	Curve fitting
*P*_PFS→PFS_	1 − *P*_PFS→PFS‐2_ − *P*_PFS→Death_	NA	NA	NA	Curve fitting
*P*_PFS‐2→PFS‐2_	1 − *P*_PFS‐2→PD_ − *P*_PFS‐2→Death_	NA	NA	NA	Curve fitting
*P*_PD→PD_	1 − *P*_PD→Death_	NA	NA	NA	Curve fitting
*P*_Death→Death_	1	NA	NA	NA	Curve fitting
Treatment cost, $					
Osimertinib (per mg)	7.08	5.31	8.85	Gamma	Medicare drug prices
Pemetrexed (per mg)	0.32	0.24	0.40	Gamma	Medicare drug prices
Cisplatin (per mg)	0.22	0.17	0.28	Gamma	Medicare drug prices
Carboplatin (per mg)	0.28	0.21	0.35	Gamma	Medicare drug prices
Paclitaxel protein bound (per mg)	14.79	11.09	18.49	Gamma	Medicare drug prices
Gefitinib (per mg)	1.09	0.82	1.36	Gamma	Medicare drug prices
Afatinib (per mg)	9.76	7.32	12.20	Gamma	Medicare drug prices
Bevacizumab (per mg)	7.38	5.54	9.23	Gamma	Medicare drug prices
Pembrolizumab (per mg)	56.41	42.31	70.51	Gamma	Medicare drug prices
Drug administration costs, $					
Chemotherapy infusion					
First 1 h	132.16	99.12	165.2	Gamma	CMS (CPT 96413)
Additional 1 h	28.47	21.35	35.59	Gamma	CMS (CPT 96415)
Per hour for subsequent infusion	65.06	48.80	81.33	Gamma	CMS (CPT 96417)
Three‐monthly imaging	114.54	85.91	143.18	Gamma	CMS (CPT 78816)
Best supportive care	3006.28	2254.71	3757.85	Gamma	Wu B, et al.^20^
End of life	40708.33	30531.25	50885.41	Gamma	Wu B, et al.^20^
Follow up	542.65	406.99	678.31	Gamma	Wu B, et al.^20^
EGFR mutation testing	1199.53	899.65	1499.41	Gamma	Wu B, et al.^20^
SAE management cost (per event), $					
Anemia	2053.86	1540.40	2567.33	Gamma	Insinga RP, et al.^21^
Neutropenia	1295.15	971.36	1618.94	Gamma	Insinga RP, et al.^21^
Platelet count decreased	2252.54	1689.41	2815.68	Gamma	Insinga RP, et al.^21^
Utility					
PFS	0.71	0.64	0.78	Beta	Chouaid C, et al.^22^
Second PFS	0.74	0.67	0.81	Beta	Chouaid C, et al.^22^
PD	0.58	0.52	0.64	Beta	Chouaid C, et al.^22^
Death	0	ND	ND	ND	Estimated
Disutility					
Anemia	0.07	0.06	0.08	Beta	Nafees B, et al.^23^
Neutropenia	0.46	0.41	0.51	Beta	Wan X, et al.^24^
Platelet count decreased	0.25	0.23	0.28	Beta	Nafees B, et al.^23^
Risk of SAEs in osimertinib group					
Anemia	0.17	0.15	0.18	Beta	Planchard D, et al.^9^
Neutropenia	0.13	0.12	0.14	Beta	Planchard D, et al.^9^
Platelet count decreased	0.07	0.06	0.08	Beta	Planchard D, et al.^9^
Risk of SAEs in osimertinib plus chemotherapy group					
Anemia	0.003	0.0027	0.0033	Beta	Planchard D, et al.^9^
Neutropenia	0.01	0.009	0.011	Beta	Planchard D, et al.^9^
Platelet count decreased	0	0	0	Beta	Planchard D, et al.^9^

Abbreviations: Ccr, creatinine clearance; CMS, Centers for Medicare & Medicaid Services; HR, hazard ratio; ND, not determined; O, osimertinib monotherapy; OC, osimertinib plus chemotherapy; OS, overall survival; PFS, profession‐free survival; SAE, severe adverse event.

^a^ Transition probabilities were time‐dependent and varied with the number of Markov cycles. *P*(t), *P*_2_(t), and S(t) were survival functions for PFS, second PFS (PFS‐2), and overall survival (OS), whose distributions and parameters were determined by curve fitting (Table S3). *P*_Natural_ was the natural modality rate per cycle derived from the World Health Organization database by a function of the “heemod” package.

### 2.4 Sensitivity analysis

To evaluate the robustness of the model, we employed both deterministic sensitivity analyses (DSA) (one‐way sensitivity analysis) and probabilistic sensitivity analyses (PSA). In the DSA, we established upper and lower limits by varying cost values, Ccr, and BSA by ±25%, and proportions and utility values by ±10%. The discount rate was set to 0.08 as the upper limit and 0 as the lower limit. In the PSA, we assigned probabilistic distributions to costs (gamma distribution), proportions (beta distribution), utility values (beta distribution), BSA (normal distribution), Ccr (normal distribution), and discount rates (uniform distribution). All model parameters were assigned suitable statistical distributions, with the mean value and standard deviation established at the baseline values and 10% of the baseline values, respectively, where applicable. A total of 1000 Monte Carlo repetitions were performed for PSA across all distributions. The ranges and distributions of model parameters were comprehensively outlined in Table 1.

### 2.5 Scenario and subgroup analyses

We took advantage of mature OS data in the osimertinib arm of the FLAURA trial, which compensated for the immature OS from the FLAURA2 trial but might lead to bias in state transitions of the base‐case Markov model. To stress this issue, we fitted original OS curves from the FLAURA2 trial with the same distribution used in the base case analysis, the gamma distribution, and reproduced the results accordingly to test the rationality of the substitution (Table S3). The time horizon was also set to 20 years (Figure S3B). In this analysis, the most recent OS curves for patients in FLAURA2 was employed.^14^

Furthermore, to investigate the heterogeneity of cost‐effectiveness between subpopulations, we carried out subgroup analyses in patients with Ex19del, patients with L858R mutations, patients with CNS metastases, and patients without CNS metastases, respectively (Figure S3C–F). The fitted PFS curves of these subgroups were used for the analyses, with other parameters remaining the same.

One‐way sensitivity analyses and probabilistic sensitivity analyses were also conducted in the scenario and subgroup analyses with the methods described above.

## 3 RESULTS

### 3.1 Base case results

The clinical characteristics of the osimertinib group in the FLAURA trial and those of the FLAURA2 trial were similar (all *p* > 0.1; Table S1), so we used the mature OS data from the FLAURA trial for the main analyses. The Markov model in the base case analysis closely imitated patients' status for 20 years (Figure S3A). The mean 10‐year cost of osimertinib plus chemotherapy was $984,917.42, while that of osimertinib was $785,941.09, indicating an incremental cost of $198,976.33. In terms of effectiveness, the mean QALYs of the osimertinib plus chemotherapy group and the osimertinib group were 3.911 and 3.022, revealing an incremental QALY of 0.8894. Based on these results, we revealed an INMB of −$65,570.70, an INHB of −0.4371 QALY at the WTP threshold of $150,000 per QALY gained, and an ICER of $223,727.1 per QALY gained, comparing osimertinib plus chemotherapy with osimertinib alone (Table 2).

**TABLE 2:** Base case results.

Treatment	Cost, $	Incremental cost, $	QALY	Incremental QALY	INMB^a^	INHB^a^	ICER ($/QALY)
Osimertinib + Chemo	984,917.42	198,976.33	3.911	0.8894	−65,570.70	−0.4371	223,727.1
Osimertinib	785,941.09	NA	3.022	NA	NA	NA	NA

^a^ At a willing‐to‐pay threshold at $150,000 per QALY gained.

Abbreviations: ICER, incremental cost‐effectiveness ratio; INHB, incremental net health benefit; INMB, incremental net monetary benefit; NA, not applicable; QALY, quality‐adjusted life year.

### 3.2 Sensitivity analyses

**FIGURE 2:**
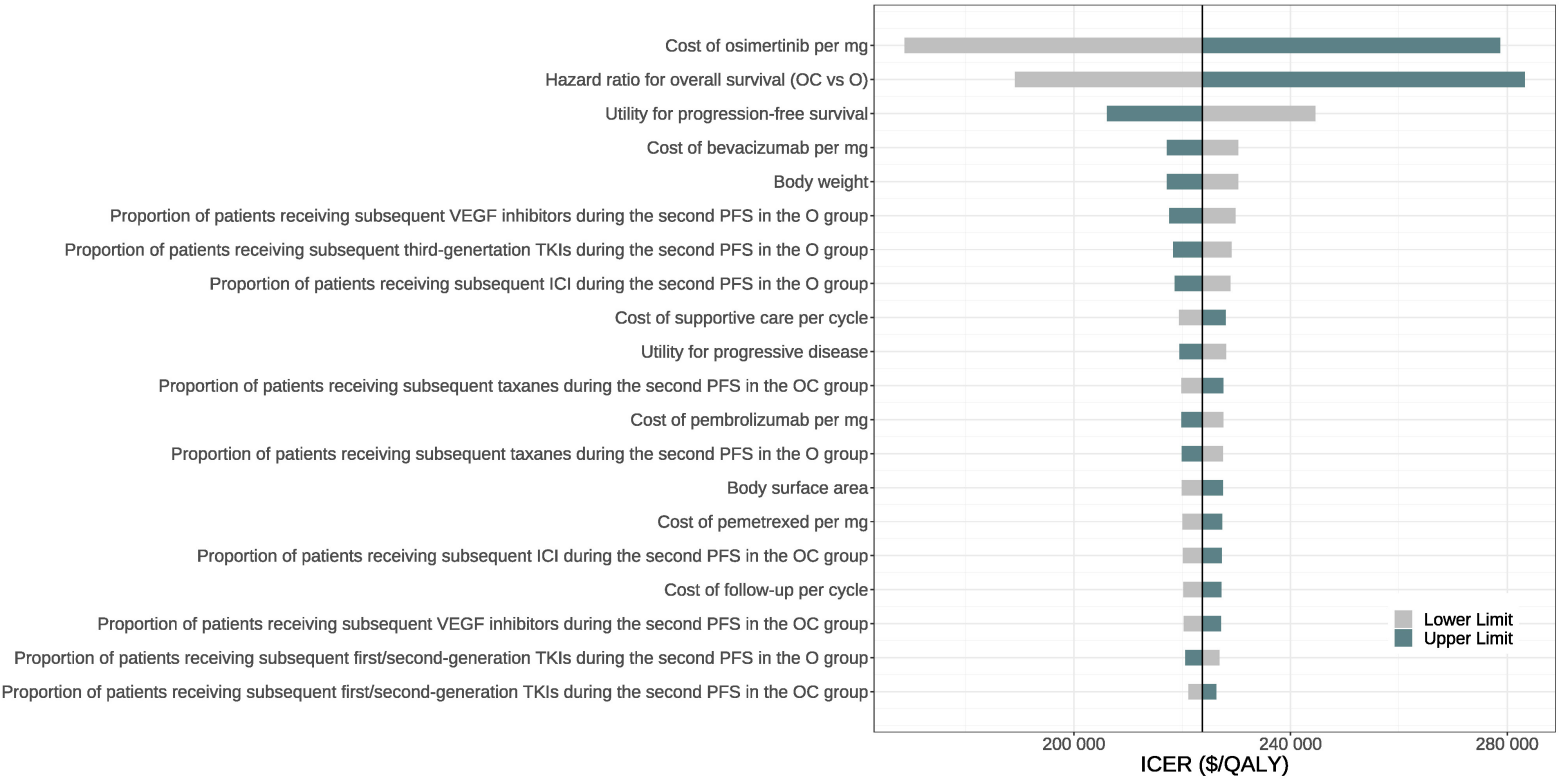
One‐way sensitivity analysis. ICER, incremental cost‐effectiveness ratio; O, osimertinib monotherapy; OC, osimertinib plus chemotherapy; PFS, progression‐free survival; QALY, quality‐adjusted life‐year; TKI, tyrosine kinase inhibitor.

Figure 2 illustrates the top 20 influential parameters in the base‐case one‐way sensitivity analysis. The tornado diagram displaying all the parameters is provided in Figure S4. When the parameters varied within the boundaries (Table 1), the ICER of osimertinib plus chemotherapy versus osimertinib alone stayed above the WTP threshold. Specifically, the result was mostly sensitive to the cost of osimertinib per mg, followed by the HR for OS. When the lower boundary ($5.31) and the upper boundary ($8.85) were considered, the ICERs were $168,761.3 and $ 278,692.9 per QALY gained, respectively. While, when the lower boundary (0.57) and the upper boundary (0.97) of HR were reached, the ICERs were $189,138.0 and $283,256.4 per QALY gained, respectively.

**FIGURE 3:**
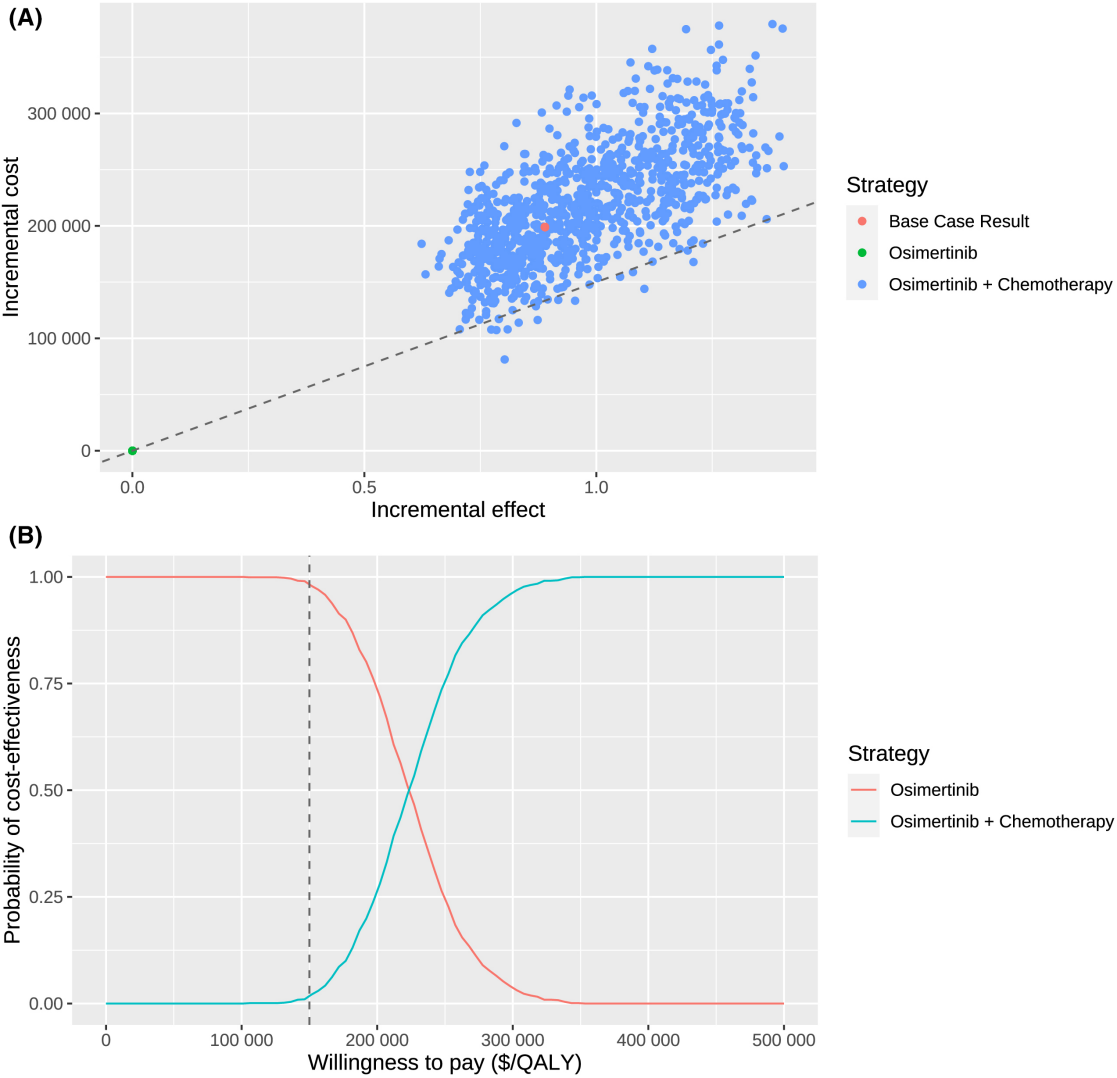
Probabilistic sensitivity analysis. (A) Incremental cost ($) and incremental effect (QALY) incurred by 1000 probabilistic resampling. (B) Probability of cost‐effectiveness at varying willingness‐to‐pay. The dashed line represents the willing‐to‐pay threshold of $150,000 per QALY gained. QALY, quality‐adjusted life‐year; WTP, willingness‐to‐pay.

The base‐case probabilistic sensitivity analysis resampling 1000 individuals demonstrated that the total costs ranged from $710,059.3 to $1,413,661.4 for osimertinib plus chemotherapy and from $614,788.1 to $1,066,803.3 for osimertinib alone. The QALYs ranged from 3.190 to 5.090 and from 2.567 to 3.689 for the two treatments, respectively. Accordingly, the ICER for osimertinib plus chemotherapy versus osimertinib ranged from $101,142.2 to $352,146.7 per QALY gained. At the WTP threshold of $150,000 per QALY gained, the probability of osimertinib plus chemotherapy being cost‐effective was only 1.9% (Figure 3).

### 3.3 Scenario analysis

Despite increases in both incremental cost ($256,948.01) and incremental QALY (1.455), the ICER revealed in this analysis, $176,608.2 per QALY gained, stayed above the WTP threshold of $150,000 per QALY gained (Table S4). The one‐way sensitivity analysis also showed that the cost of osimertinib per mg was the most influential parameter (Figure S5A), and the probabilistic sensitivity analysis revealed a 16.8% probability of osimertinib plus chemotherapy being cost‐effective at the WTP threshold (Figure S5B,C).

### 3.4 Subgroup analyses

The results of the subgroup analyses were summarized in Table 3. As for patients with the L858R mutation, the costs of osimertinib plus chemotherapy and osimertinib alone were respectively $806,728.20and $725,843.12, and the QALYs of the two treatments were respectively 3.405 and 2.795, yielding an ICER of $132,614.1 per QALY gained, which was below the WTP threshold. On the contrary, in patients with Ex19del, the costs were respectively $968,788.06 and $761,504.39, while the QALYs were respectively 3.866 and 2.942, bringing about a much higher ICER of $224,449.8 per QALY gained than that of the L858R subgroup.

**TABLE 3:** Subgroup analyses.

Treatment	Cost, $	Incremental cost, $	QALY	Incremental QALY	INMB^a^	INHB^a^	ICER ($/QALY)
L858R							
Osimertinib + Chemo	806,728.20	80,885.08	3.405	0.6099	10,604.17	0.07069	132,614.1
Osimertinib	725,843.12	NA	2.795	NA	NA	NA	NA
Ex19del							
Osimertinib + Chemo	968,788.06	207,283.67	3.866	0.9235	−68,755.83	−0.4584	224,449.8
Osimertinib	761,504.39	NA	2.942	NA	NA	NA	NA
CNSm							
Osimertinib + Chemo	892,259.29	188,890.46	3.649	0.9376	−48,252.13	−0.3217	201,464.1
Osimertinib	703,368.83	NA	2.712	NA	NA	NA	NA
No CNSm							
Osimertinib + Chemo	844,823.54	67,081.55	3.514	0.5154	10,225.28	0.06817	130,159.7
Osimertinib	777,741.99	NA	2.999	NA	NA	NA	NA

^a^ At a willing‐to‐pay threshold at $150,000 per QALY gained.

Abbreviations: ICER, incremental cost‐effectiveness ratio; INHB, incremental net health benefit; INMB, incremental net monetary benefit; NA, not applicable; QALY, quality‐adjusted life year.

In terms of patients with CNS metastases, the costs of osimertinib plus chemotherapy and osimertinib alone were respectively $892,259.29 and $703,368.83, and the QALYs were 3.649 and 2.712 for the two groups, respectively, revealing an ICER of $201,464.1 per QALY gained. In contrast, in patients without CNS metastases, the costs were respectively $844,823.54 and $777,741.99, and the QALYs were respectively 3.514 and 2.999, generating a much lower ICER of $130,159.7 per QALY gained than that of the CNS metastases‐positive subgroup.

All four subgroup one‐way sensitivity analyses identified the HR for OS and the cost of osimertinib per mg as the top two influential parameters determining the cost‐effectiveness (Figure S6). As for the probabilistic sensitivity analyses, the probabilities of osimertinib plus chemotherapy being cost‐effective were 63.5%, 1.9%, 1.04%, and 67.6% for patients with the L858R mutation, those with the Ex19del mutation, those with CNS metastases, and those without CNS metastases, respectively (Figure S7).

## 4 DISCUSSION

For treatment‐naïve patients with advanced NSCLC and EGFR Ex19del or L858R mutations, osimertinib is the preferred therapy, according to the NCCN guideline. Recently, the results from the FLAURA2 trial demonstrated that the combination of osimertinib and chemotherapy significantly extended patients' PFS (HR = 0.62; 95% CI 0.48–0.80) and second PFS (HR = 0.7; 95% CI 0.52–0.93) compared with osimertinib alone.^9^ The latest interim analysis also showed that osimertinib plus chemotherapy prolonged OS of the patients compared with osimertinib monotherapy (HR = 0.75; 95% CI 0.57–0.97).^14^ However, the combination therapy was accompanied by not only higher costs on chemotherapy regimens but also a higher rate of severe AEs, which led to considerable costs for AE management and decreases in patients' quality of life. Encouraged by this observation, we designed and conducted this study, and the results showed that osimertinib plus chemotherapy was not cost‐effective in the whole population but was cost‐effective in patients with the L858R mutation and patients without CNS metastases at the WTP threshold of $150,000 per QALY gained.

Based on results from the FLAURA and AURA3 trials, efforts have been made to evaluate the cost‐effectiveness of osimertinib monotherapy in EGFR‐mutated (Ex19del or L858R) patients with advanced NSCLC. In 2018, a study showed that the ICER of osimertinib versus other EGFR‐TKIs (gefitinib, erlotinib, or afatinib) ranged from $219,874 to $231,123 per QALY gained in the United States, while the ICER in Brazil ranged from $162,329 to $180,804 per QALY gained, indicating osimertinib was not cost‐effective in neither of the two countries.^28^ A similar study showed that osimertinib was not cost‐effective in China, either, with an ICER of $39,369.53/QALY, and the cost of osimertinib offered the primary influence on the results.^29^ In another study, the researchers evaluated the cost‐effectiveness of 12 first‐line treatments, including osimertinib monotherapy, for patients with advanced EGFR‐mutated NSCLC from the perspectives of the United Kingdom and China.^30^ The results showed that gefitinib and gefitinib plus chemotherapy dominated chemotherapy in both countries, while osimertinib was not cost‐effective versus chemotherapy in neither the United Kingdom (ICER = £1,269,085/QALY) nor China (ICER = ¥224,999).^30^ In terms of second‐line settings, a study in 2019 compared the cost‐effectiveness of first‐line osimertinib, second‐line osimertinib (after progression on prior EGFR‐TKIs), and control (erlotinib or gefitinib) in both the United States and China.^31^ The ICER of first‐line osimertinib vs control and that of second‐line osimertinib versus control were respectively $312,903 and $284,532 per QALY gained in the United States, and those in China were $41,512 and $38,860 per QALY, respectively, all of which were above the WTP thresholds.^31^ Their deterministic sensitivity analysis revealed the cost of osimertinib as the primary determinant.^31^ A more focused comparison between second‐line osimertinib and chemotherapy in another study showed that osimertinib was not cost‐effective in either the United States (ICER = $232,895/QALY) or China (ICER = $239,274/QALY).^20^ However, another study showed that second‐line osimertinib for patients with EGFR‐T790M mutations was cost‐effective in the United Kingdom (ICER = £41,705/QALY), compared with chemotherapy, although the probability of its cost‐effectiveness was moderate (63.4%).^32^ In all, osimertinib, although effective, is not economically friendly for patients with EGFR‐mutated patients with advanced NSCLC, but decreasing its price might make it more favorable in cost‐effectiveness, especially for patients with T790M mutations. Based on these results, it would be reasonable to assume that osimertinib plus chemotherapy may exhibit better cost‐effectiveness in second‐line settings for patients with T790M than in first‐line settings.

In line with previous studies, our analyses added that first‐line osimertinib plus chemotherapy was not cost‐effective for EGFR‐mutated advanced NSCLC patients, either. The deterministic sensitivity analyses supported that the cost of osimertinib per mg and the HR for OS were two primary factors influencing the final economic results, suggesting that cutting down the price of osimertinib is an effective way to improve its cost‐effectiveness and that the final OS data with a longer follow‐up is essential to conclude. Besides, the probabilistic sensitivity analyses approved the robustness of the results in the total population, patients with Ex19del, and patients with CNS metastases. Although first‐line osimertinib plus chemotherapy is not cost‐effective in the whole EGFR‐mutated (Ex19del or L858R) advanced NSCLC population, our subgroup analyses have revealed heterogeneities among populations. First, patients with the L858R mutation have a significantly lower ICER ($132,614.1 per QALY gained) than patients with Ex19del do ($224,449.8 per QALY gained). Second, patients without CNS metastases had a lower ICER ($130,159.7 per QALY gained) than patients with CNS metastases ($201,464.1 per QALY gained). These are two reasonable observations because patients with Ex19del (OS‐HR = 0.68; 95% CI 0.51–0.90) or patients without CNS metastases (OS‐HR = 0.79; 95% CI 0.61–1.01) tend to respond more distinguishingly to osimertinib than those with the L858R (OS‐HR = 1.00; 95% CI 0.71–1.40) or those with CNS metastases (OS‐HR = 0.83; 95% CI 0.53–1.30) comparing with other EGFR‐TKIs.^7^ Thus, the addition of chemotherapy may compensate for efficacy defect in these two population and improve patients' QALY more evidently than others, which is supported by the PFS data from the FLAURA2 trial.^9^

This study has several limitations. First, because the OS data of the FLAURA2 trial were premature (data maturity, 41%),^14^ we alternatively used the mature OS from the FLAURA trial to estimate the survival probability of patients receiving osimertinib and subsequently calculated the survival probability of the other arm with the HR for OS. This substitution, although reasonable, may still cause inaccuracy in the results. Accordingly, we also fitted the latest FLAURA2 OS curves to reproduce the results, which also showed a lack of cost‐effectiveness of osimertinib. Second, it is notable that the results are distribution‐dependent due to the short follow‐up. Specifically, when different distributions are chosen to fit the survival curves, the cost‐effectiveness results vary drastically. To minimize its effect, we unified distributions for the same measurements (the gamma distribution for PFS and OS; and the Gompertz distribution for second PFS) in all analyses. Third, we used OS and second PFS data of the whole population to imitate patients' survival in the 4 subgroups, as the OS data of each group were unavailable. This estimation could cause bias in subgroup analysis because patients with Ex19del and patients without CNS metastases are likely to have longer OS and second PFS than those with L858R mutations and those with CNS metastases, respectively.

## 5 CONCLUSIONS

From the perspective of the United States health care system, first‐line osimertinib plus chemotherapy is not cost‐effective compared with osimertinib monotherapy for patients with EGFR‐mutated (Ex19del or L858R mutations) advanced NSCLC. However, more favorable cost‐effectiveness occurs in patients with L858R mutations and patients without CNS metastases.

## AUTHOR CONTRIBUTIONS

**Wentao Tian:** Data curation (equal); formal analysis (equal); methodology (equal); resources (equal); software (equal); visualization (equal); writing – original draft (equal). **Lishui Niu:** Data curation (equal); formal analysis (equal); methodology (equal); resources (equal); software (equal); visualization (equal); writing – original draft (equal). **Rongrong Zhou:** Conceptualization (equal); funding acquisition (equal); project administration (equal); resources (equal); writing – review and editing (equal). **Ziqi Wang:** Data curation (equal); writing – review and editing (equal). **Ruoyu Lu:** Data curation (equal); writing – review and editing (equal). **Jiaoyang Ning:** Data curation (equal). **Yin Shi:** Writing – review and editing (equal). **Zhaohua Tan:** Conceptualization (equal); writing – review and editing (equal).

## FUNDING INFORMATION

This study was supported by the National Multidisciplinary Cooperative Diagnosis and Treatment Capacity Building Project for Major Diseases (Lung Cancer, grant number: z027002), Natural Science Foundation of Hunan Province (2022JJ30992), Chen Xiao‐Ping Foundation for the Development of Science and Technology of Hubei Province (CXPJJH121005‐01).

## CONFLICT OF INTEREST STATEMENT

The authors declare no conflict of interest concerning this work.

## PRÉCIS

Osimertinib plus chemotherapy is not cost‐effective compared to osimertinib alone for treatment‐naïve patients with EGFR‐mutated advanced non‐small cell lung cancer in the United States. More favorable cost‐effectiveness occurs in patients with L858R mutations and patients without baseline central nervous system metastases.

## Supporting information


Data S1.

## Data Availability

The data that support the findings of this study will be openly available in FLAURA2‐CEA at https://github.com/TwT9807/FLAURA2‐CEA.git, reference number not available.
