# Supplementary material for: Cost‐effectiveness analysis of osimertinib plus chemotherapy for patients with EGFR‐mutated advanced non‐small cell lung cancer
Source: Cancer Med. 2024 Aug 29;13(16):e70083. doi: 10.1002/cam4.70083 (PMC11358701; doi:10.1002/cam4.70083)
Supplement: Supplementary file 1 — Data S1. [file CAM4-13-e70083-s001.docx]

Supplementary Material

**Table S1.** Characteristics of populations in the osimertinib arms of the FLAURA2 trial and the FLAURA trial.

| **Characteristics** | **FLAURA2 (N=278)** | **FLAURA(N=279)** | **p** |
| --- | --- | --- | --- |
| **Age — year** |  |  | 1.0 |
| Median | 62 | 64 |  |
| Range | 30-85 | 26-85 |  |
| **Sex — no. (%)** |  |  | 0.52 |
| Male | 109 (39) | 101 (36) |  |
| Female | 169 (61) | 178 (64) |  |
| **Race — no. (%)** |  |  | 0.89 |
| Asian | 176 (63) | 174 (62) |  |
| Others | 102 (37) | 105 (38) |  |
| **WHO performance-status score — no. (%)** |  |  | 0.45 |
| 0 | 102 (37) | 112 (40) |  |
| 1 | 176 (63) | 167 (60) |  |
| **EGFR mutation status — no. (%)** |  |  | 0.20 |
| Exon 19 deletion | 168 (60) | 175 (63) |  |
| L858R mutation | 107 (38) | 104 (37) |  |
| Both Exon 19 deletion and L858R mutation or unknown | 3 (2) | 0 |  |
| **Overall disease classification — no. (%)** |  |  | 0.18 |
| Metastatic | 271 (97) | 264 (95) |  |
| Locally advanced | 7 (3) | 14 (5) |  |
| Missing data | 0 | 1 (<1) |  |
| **Histologic type — no. (%)** |  |  | 1.0 |
| Adenocarcinoma | 275 (99) | 275 (99) |  |
| Others | 3 (1) | 4 (1) |  |

**Table S2.** Summary of AIC and BIC Scores for Parametric Models.

| Scores | Exponential | Weibull | Loglogistic | Lognormal | Gengamma | Gamma | Gompertz |
| --- | --- | --- | --- | --- | --- | --- | --- |
| OS of the osimertinib arm (FLAURA) | | | | | | | |
| AIC | 1555.60 | 1515.33 | 1517.30 | 1520.47 | 1517.08 | 1515.16 | 1523.27 |
| BIC | 1559.24 | 1522.59 | 1524.57 | 1527.74 | 1527.98 | 1522.42 | 1530.53 |
| OS of the osimertinib plus chemotherapy arm (FLAURA2) | | | | | | | |
| AIC | 1077.91 | 1076.54 | 1081.41 | 1093.35 | 1074.29 | 1077.57 | 1070.17 |
| BIC | 1081.54 | 1083.81 | 1088.67 | 1100.61 | 1085.18 | 1084.83 | 1077.44 |
| OS of the osimertinib arm (FLAURA2) | | | | | | | |
| AIC | 1290.98 | 1260.25 | 1264.59 | 1280.69 | 1260.68 | 1263.56 | 1259.10 |
| BIC | 1294.60 | 1267.50 | 1271.85 | 1287.95 | 1271.56 | 1270.81 | 1266.35 |
| PFS of the osimertinib plus chemotherapy arm | | | | | | | |
| AIC | 1001.65 | 1001.56 | 1004.42 | 1013.47 | 1002.16 | 1001.97 | 1000.09 |
| BIC | 1005.28 | 1008.83 | 1011.68 | 1020.73 | 1013.05 | 1009.23 | 1007.35 |
| PFS of the osimertinib arm | | | | | | | |
| AIC | 1235.71 | 1232.87 | 1230.34 | 1230.42 | 1231.55 | 1231.92 | 1236.38 |
| BIC | 1239.34 | 1240.12 | 1237.59 | 1237.68 | 1242.43 | 1239.18 | 1243.63 |
| Second PFS of the osimertinib plus chemotherapy arm | | | | | | | |
| AIC | 843.56 | 839.28 | 844.23 | 857.21 | 836.21 | 841.06 | 828.47 |
| BIC | 847.19 | 846.55 | 851.50 | 864.48 | 847.10 | 848.32 | 835.73 |
| Second PFS of the osimertinib arm | | | | | | | |
| AIC | 1023.90 | 994.15 | 997.71 | 1010.82 | 994.75 | 997.05 | 993.37 |
| BIC | 1027.53 | 1001.40 | 1004.96 | 1018.08 | 1005.63 | 1004.31 | 1000.63 |
| PFS of the osimertinib plus chemotherapy arm in patients with L858R mutations | | | | | | | |
| AIC | 493.89 | 486.25 | 488.42 | 492.48 | 487.80 | 487.02 | 486.47 |
| BIC | 496.55 | 491.57 | 493.75 | 497.81 | 495.79 | 492.34 | 491.80 |
| PFS of the osimertinib arm in patients with L858R mutations | | | | | | | |
| AIC | 577.25 | 577.17 | 577.81 | 581.06 | 578.93 | 576.99 | 578.18 |
| BIC | 579.92 | 582.51 | 583.15 | 586.40 | 586.94 | 582.34 | 583.53 |
| PFS of the osimertinib plus chemotherapy arm in patients with Ex19del | | | | | | | |
| AIC | 638.03 | 637.39 | 640.92 | 649.99 | 636.59 | 638.18 | 633.67 |
| BIC | 641.18 | 643.68 | 647.22 | 656.29 | 646.03 | 644.47 | 639.97 |
| PFS of the osimertinib arm in patients with Ex19del | | | | | | | |
| AIC | 856.42 | 846.83 | 846.90 | 848.73 | 848.17 | 846.29 | 850.93 |
| BIC | 859.55 | 853.09 | 853.16 | 854.99 | 857.56 | 852.55 | 857.19 |
| PFS of the osimertinib plus chemotherapy arm in patients with CNS metastases | | | | | | | |
| AIC | 483.32 | 482.06 | 485.63 | 493.11 | 481.83 | 483.01 | 478.89 |
| BIC | 486.07 | 487.57 | 491.13 | 498.62 | 490.09 | 488.52 | 484.39 |
| PFS of the osimertinib arm in patients with CNS metastases | | | | | | | |
| AIC | 636.30 | 632.50 | 631.06 | 631.35 | 632.40 | 631.53 | 635.99 |
| BIC | 639.00 | 637.90 | 636.46 | 636.75 | 640.50 | 636.93 | 641.39 |
| PFS of the osimertinib plus chemotherapy arm in patients without CNS metastases | | | | | | | |
| AIC | 689.09 | 676.19 | 682.46 | 691.22 | 671.19 | 678.75 | 669.68 |
| BIC | 692.18 | 682.38 | 688.65 | 697.41 | 680.48 | 684.94 | 675.87 |
| PFS of the osimertinib arm in patients without CNS metastases | | | | | | | |
| AIC | 813.21 | 806.45 | 808.21 | 812.61 | 808.45 | 806.60 | 808.07 |
| BIC | 816.34 | 812.70 | 814.46 | 818.86 | 817.83 | 812.85 | 814.32 |

**Table S3.** Parameters of fitted distributions in the base-case, scenario, and subgroup analyses.

| Parameters | Baseline Value | Range | | Distribution | Source |
| --- | --- | --- | --- | --- | --- |
|  |  | Minimum | Maximum |  |  |
| **Base-case Analysis** | | | | | |
| Gamma OS survival model of the O arm in the FLAURA trial | shape: 2.075 | ND | ND | ND | Model fitting |
|  | rate: 0.04584 |  |  |  |  |
| Gamma PFS survival model of the O arm | shape: 1.285 | ND | ND | ND | Model fitting |
|  | rate: 0.04602 |  |  |  |  |
| Gamma PFS survival model of the OC arm | shape: 1.161 | ND | ND | ND | Model fitting |
|  | rate: 0.02599 |  |  |  |  |
| Gompertz second PFS survival model of the O arm | shape: 0.07052 | ND | ND | ND | Model fitting |
|  | rate: 0.007330 |  |  |  |  |
| Gompertz second PFS survival model of the OC arm | shape: 0.05917 | ND | ND | ND | Model fitting |
|  | rate: 0.006341 |  |  |  |  |
| **Scenario Analyses** | | | | | |
| Gamma OS survival model of the OC arm in the FLAURA2 trial | shape: 1.197 | ND | ND | ND | Model fitting |
|  | rate: 0.01704 |  |  |  |  |
| Gamma OS survival model of the O arm in the FLAURA2 trial | shape: 1.914 | ND | ND | ND | Model fitting |
|  | rate: 0.04160 |  |  |  |  |
| **L858R Subgroup** |  |  |  |  |  |
| Gamma PFS survival model of the OC arm | shape: 1.699 | ND | ND | ND | Model fitting |
|  | rate: 0.05900 |  |  |  |  |
| Gamma PFS survival model of the O arm | shape: 1.255 | ND | ND | ND | Model fitting |
|  | rate: 0.05953 |  |  |  |  |
| **Ex19del Subgroup** |  |  |  |  |  |
| Gamma PFS survival model of the OC arm | shape: 1.219 | ND | ND | ND | Model fitting |
|  | rate: 0.02822 |  |  |  |  |
| Gamma PFS survival model of the O arm | shape: 1.594 | ND | ND | ND | Model fitting |
|  | rate: 0.06163 |  |  |  |  |
| **CNS Metastases Subgroup** |  |  |  |  |  |
| Gamma PFS survival model of the OC arm | shape: 1.292 | ND | ND | ND | Model fitting |
|  | rate: 0.03566 |  |  |  |  |
| Gamma PFS survival model of the O arm | shape: 1.475 | ND | ND | ND | Model fitting |
|  | rate: 0.07837 |  |  |  |  |
| **No CNS Metastases Subgroup** |  |  |  |  |  |
| Gamma PFS survival model of the OC arm | shape: 1.690 | ND | ND | ND | Model fitting |
|  | rate: 0.05241 |  |  |  |  |
| Gamma PFS survival model of the O arm | shape: 1.485 | ND | ND | ND | Model fitting |
|  | rate: 0.05406 |  |  |  |  |

***Abbr.*** *OC, osimertinib plus chemotherapy; O, osimertinib monotherapy; CNS, central nervous system; PFS, profession-free survival; ND, not determined.*

**Table S4.** Scenario analysis using original OS curves from the FLAURA2 trial.

| **Treatment** | **Cost, $** | **Incremental Cost, $** | **QALY** | **Incremental QALY** | **INMB*** | **INHB*** | **ICER ($/QALY)** |
| --- | --- | --- | --- | --- | --- | --- | --- |
| Osimertinib + Chemo | 1,047,832.82 | 256,948.01 | 4.524 | 1.455 | -38712.40 | -0.2581 | 176,608.2 |
| Osimertinib | 790,884.81 | NA | 3.069 | NA | NA | NA | NA |

*At a willing-to-pay threshold at $150,000 per QALY gained.

***Abbr.*** *QALY = quality-adjusted life year; INMB = incremental net monetary benefit; INHB: incremental net health benefit; ICER = incremental cost-effectiveness ratio; NA: not applicable.*


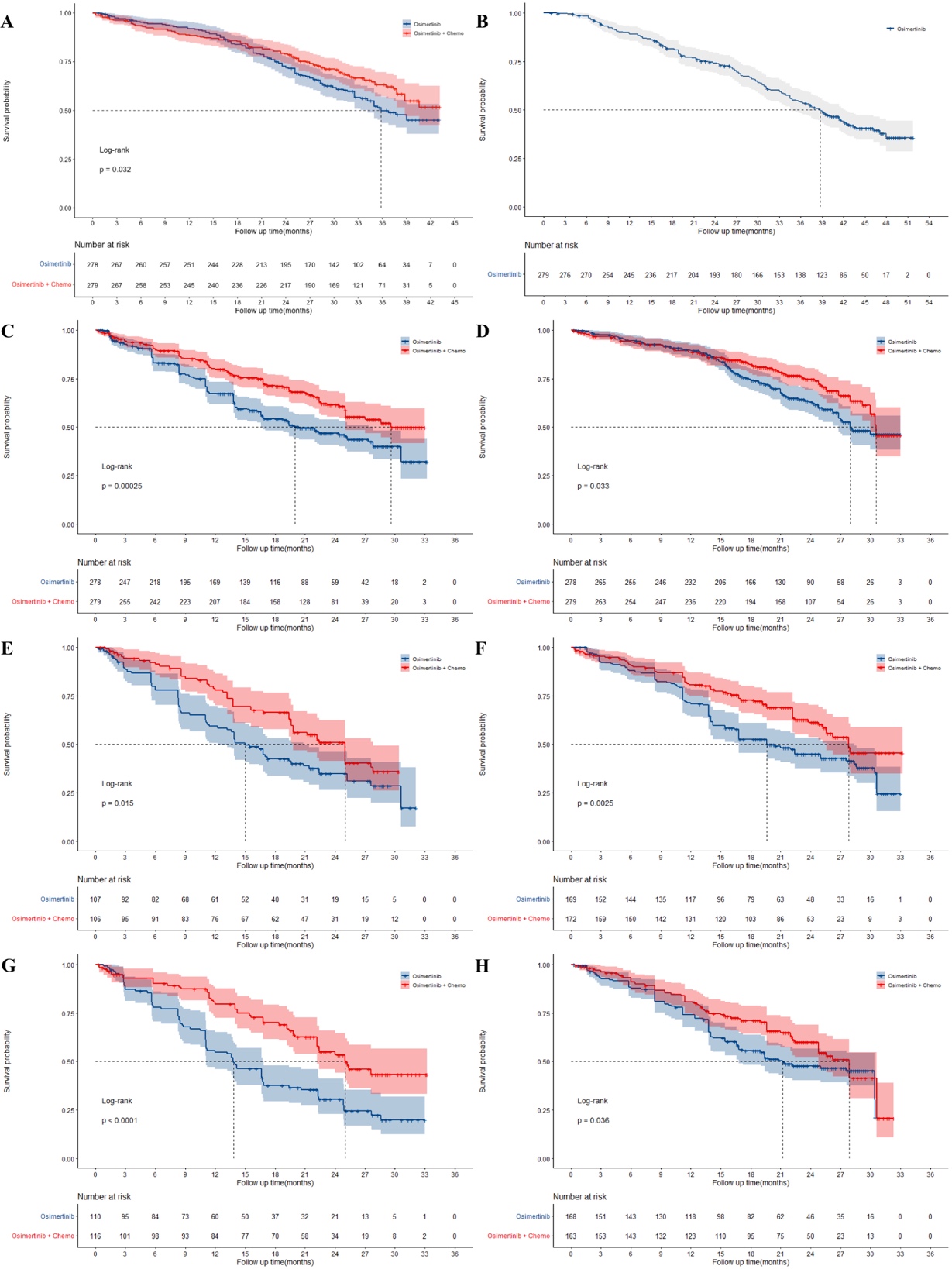


**Figure S1.** Replicated Kaplan-Meier Curves. A) OS; B) OS of the osimertinib arm in the FLAURA trial; C) PFS; D) second PFS; E) PFS of patients with the L858R mutations; F) PFS of patients with Ex19del; G) PFS of patients with CNS metastases; H) Patients without CNS metastases. ***Abbr.*** *OS, overall survival; PFS, progression-free survival; Ex19del, exon 19 deletion; CNS, central nervous system.*


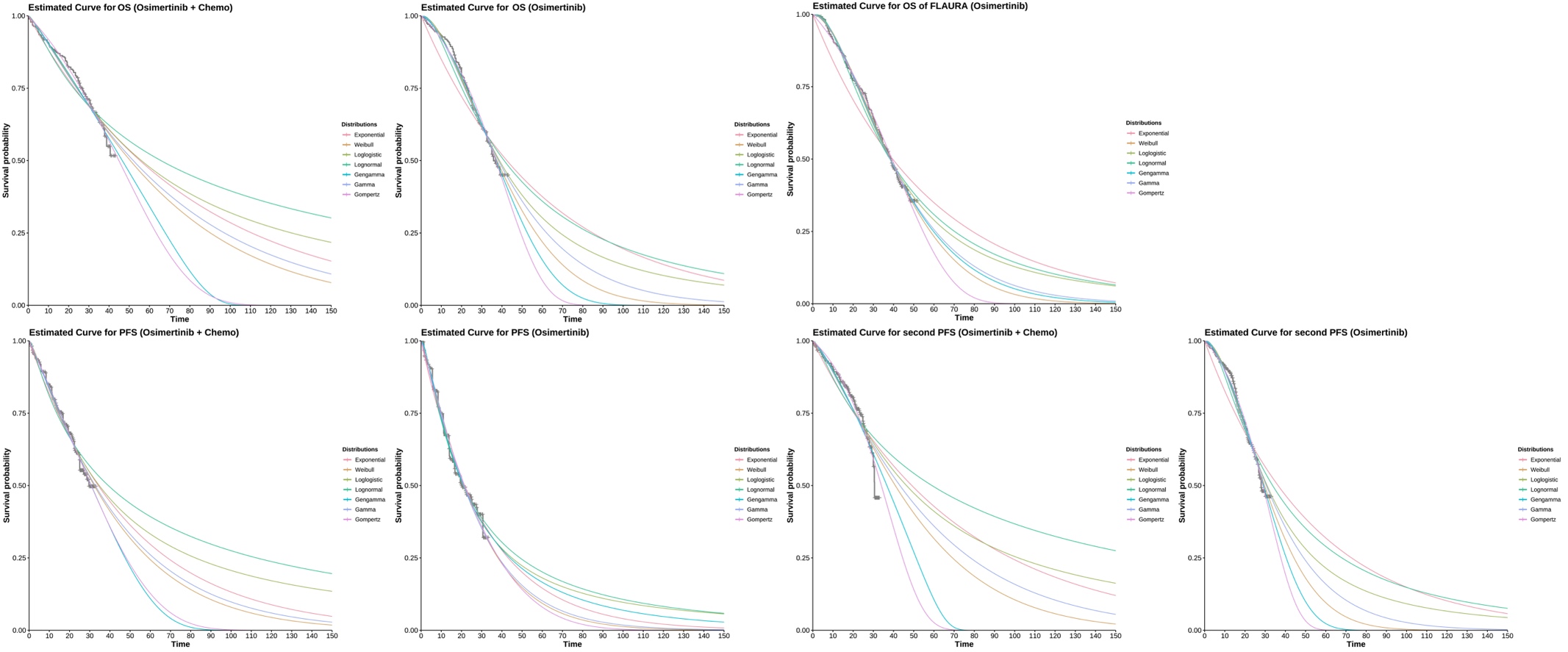

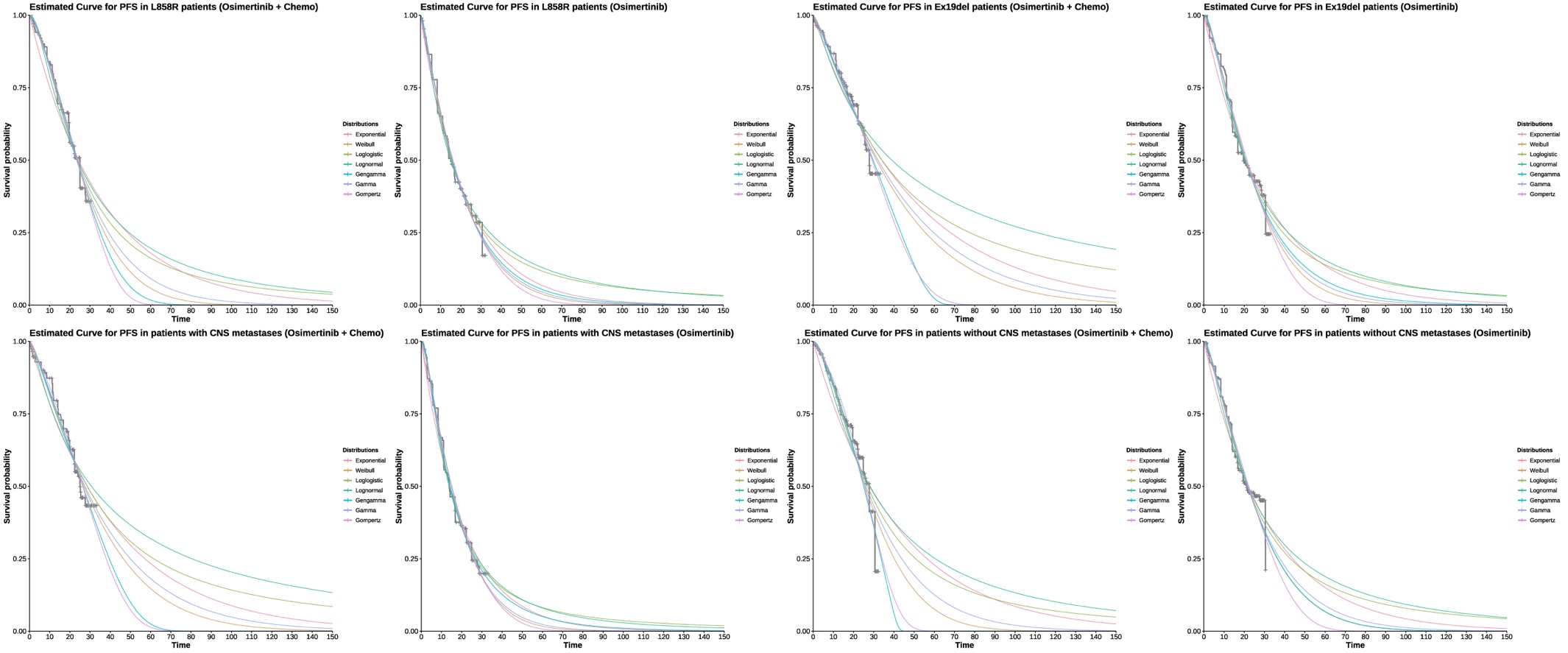


**Figure S2.** Estimated Curves using exponential, Weibull, lognormal, log-logistic, generalized gamma, gamma, and Gompertz distributions.


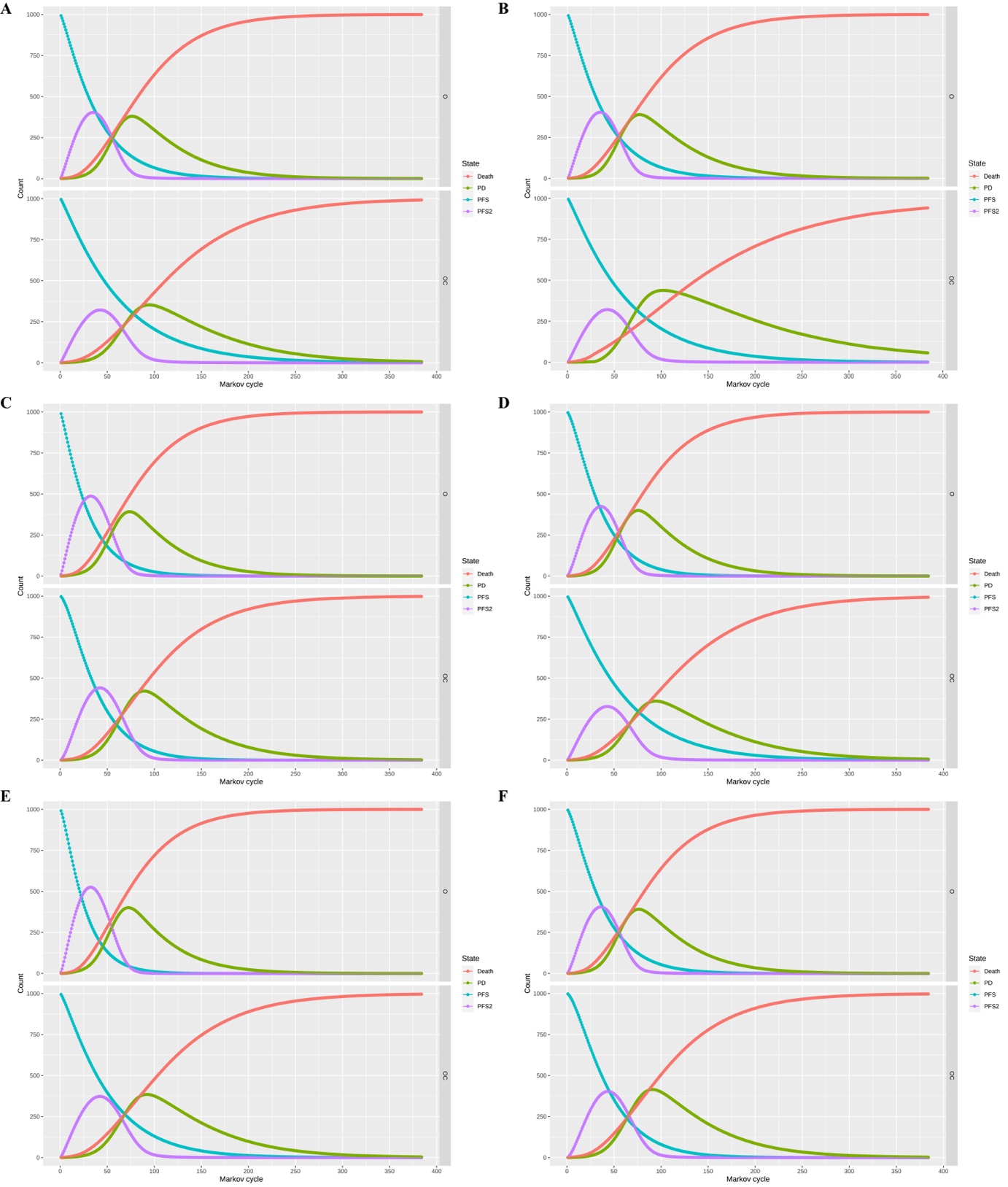


**Figure S3.** Patient counts in 21-day Markov cycles for 20 years. A) Base case analysis; B) Scenario analysis using the original OS curves from the FLAURA2 trial; C) Analysis in patients with the L858R mutations; D) Analysis in patients with Ex19del; E) Analysis in patients with CNS metastases; F) Analysis in patients without CNS metastases.


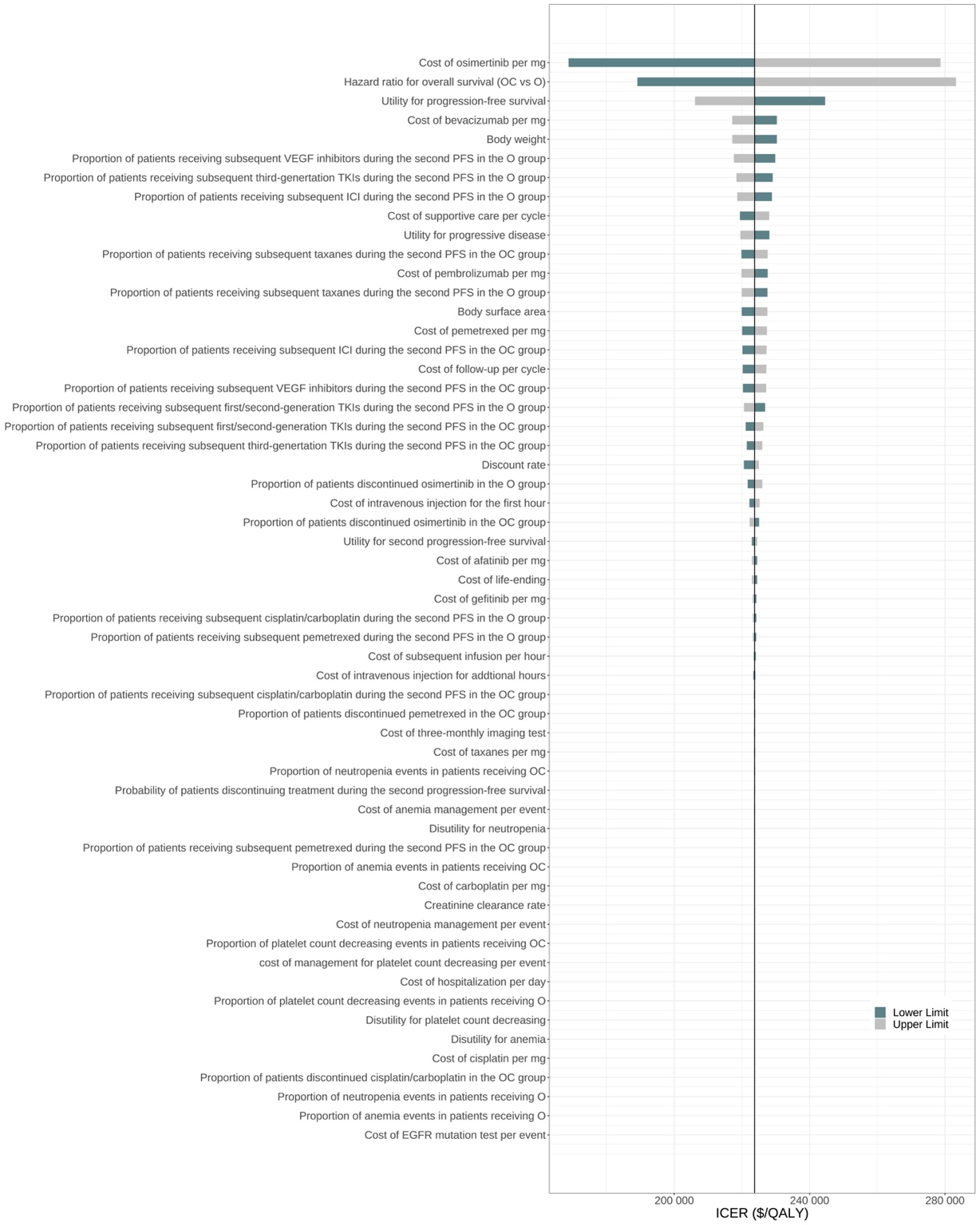


**Figure S4.** Full Tornado Diagram of the One-way Sensitivity Analysis. ***Abbr.*** *ICER, incremental cost-effectiveness ratio; QALY, quality-adjusted life-year; TKI, tyrosine kinase inhibitor; OC, osimertinib plus chemotherapy; O, osimertinib monotherapy; PFS, progression-free survival.*


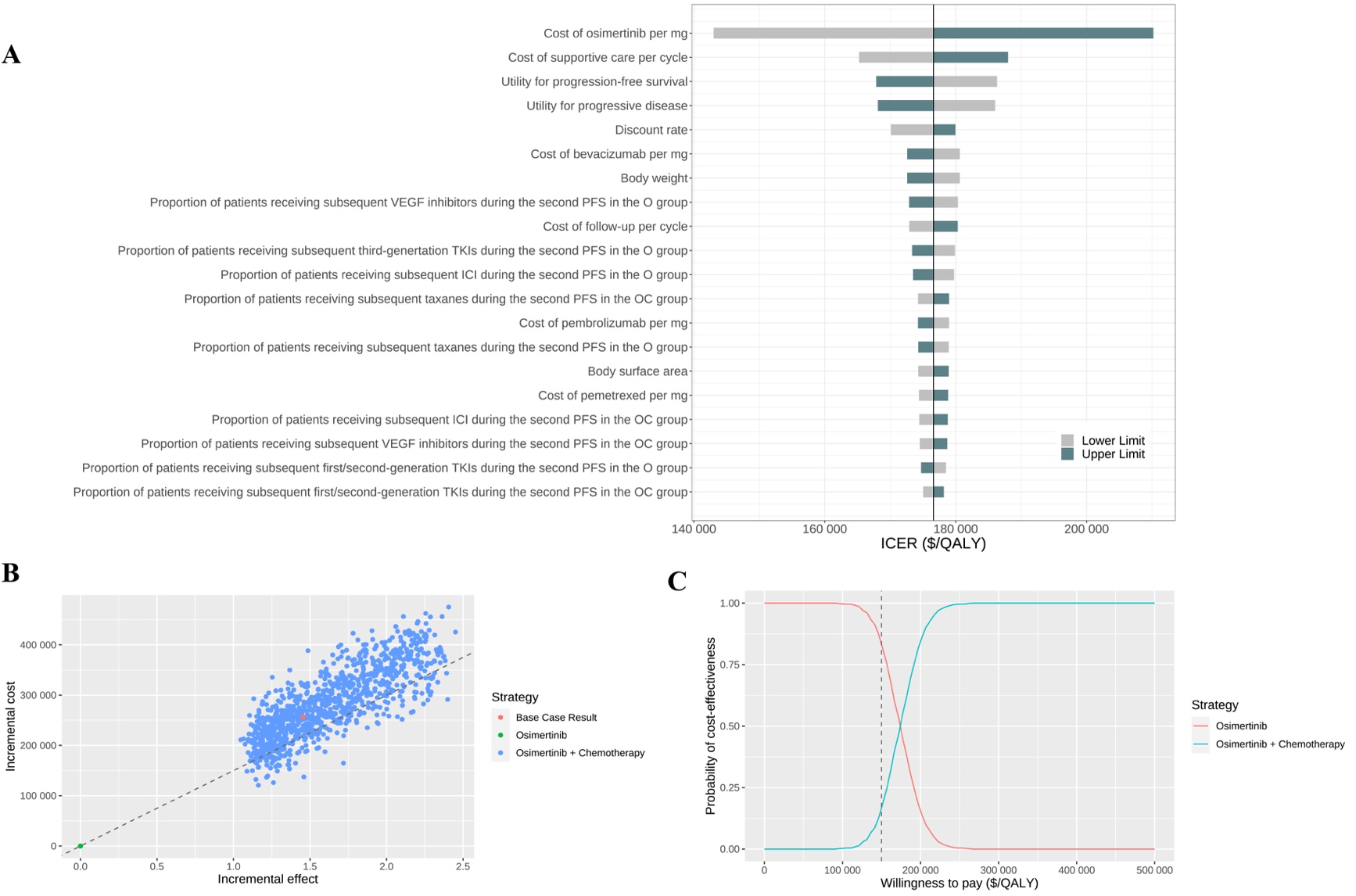


**Figure S5.** Sensitivity Analyses of the Scenario Analysis. A) One-way sensitivity analysis; B) Incremental cost ($) and incremental effect (QALY) incurred by 1,000 probabilistic resamples in the probabilistic sensitivity analysis; C) Probability of cost-effectiveness at varying WTP. The dashed line represents the WTP threshold of $150,000 per QALY gained. ***Abbr.*** *ICER, incremental cost-effectiveness ratio; QALY, quality-adjusted life-year; TKI, tyrosine kinase inhibitor; OC, osimertinib plus chemotherapy; O, osimertinib monotherapy; PFS, progression-free survival.*


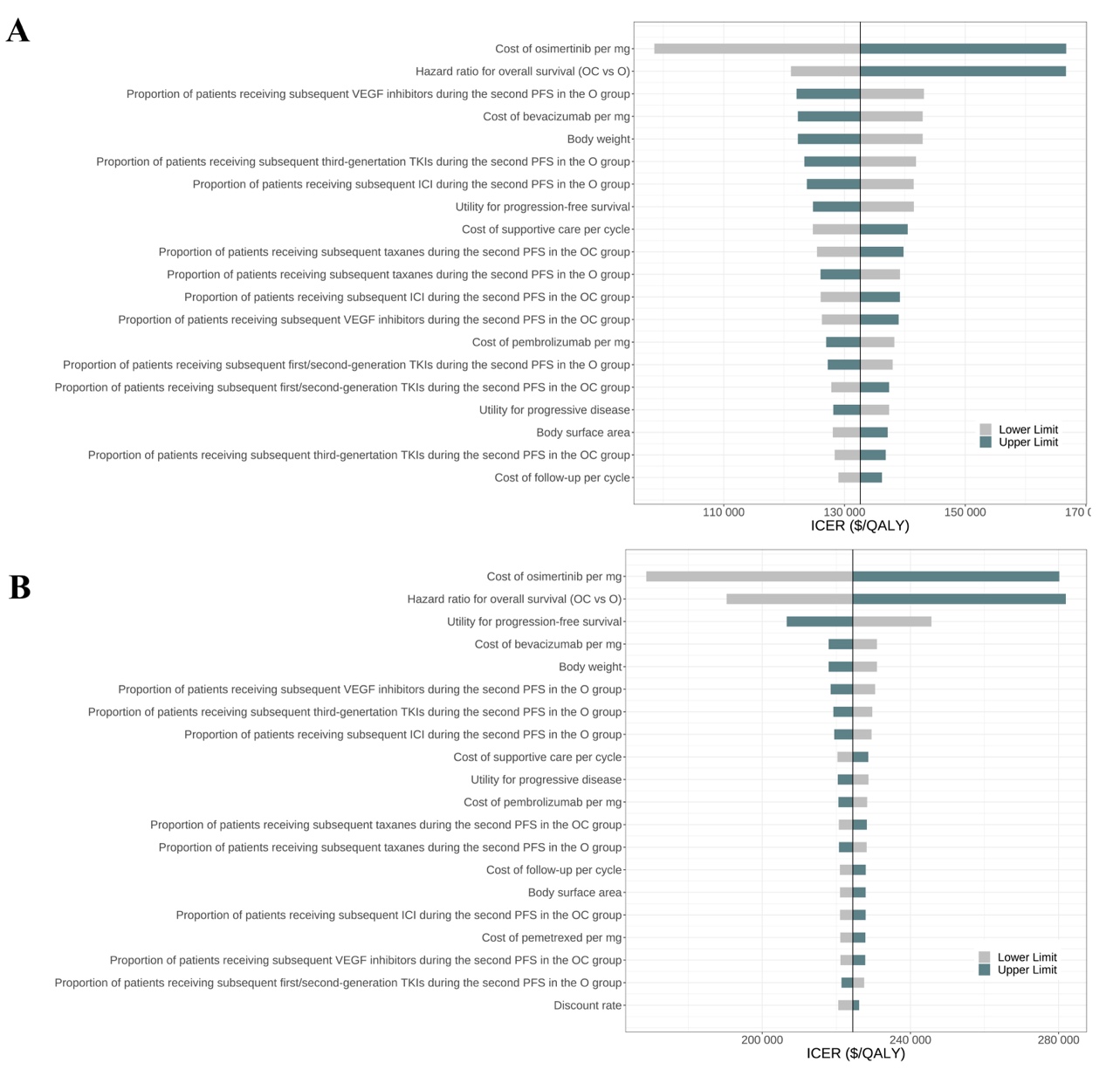

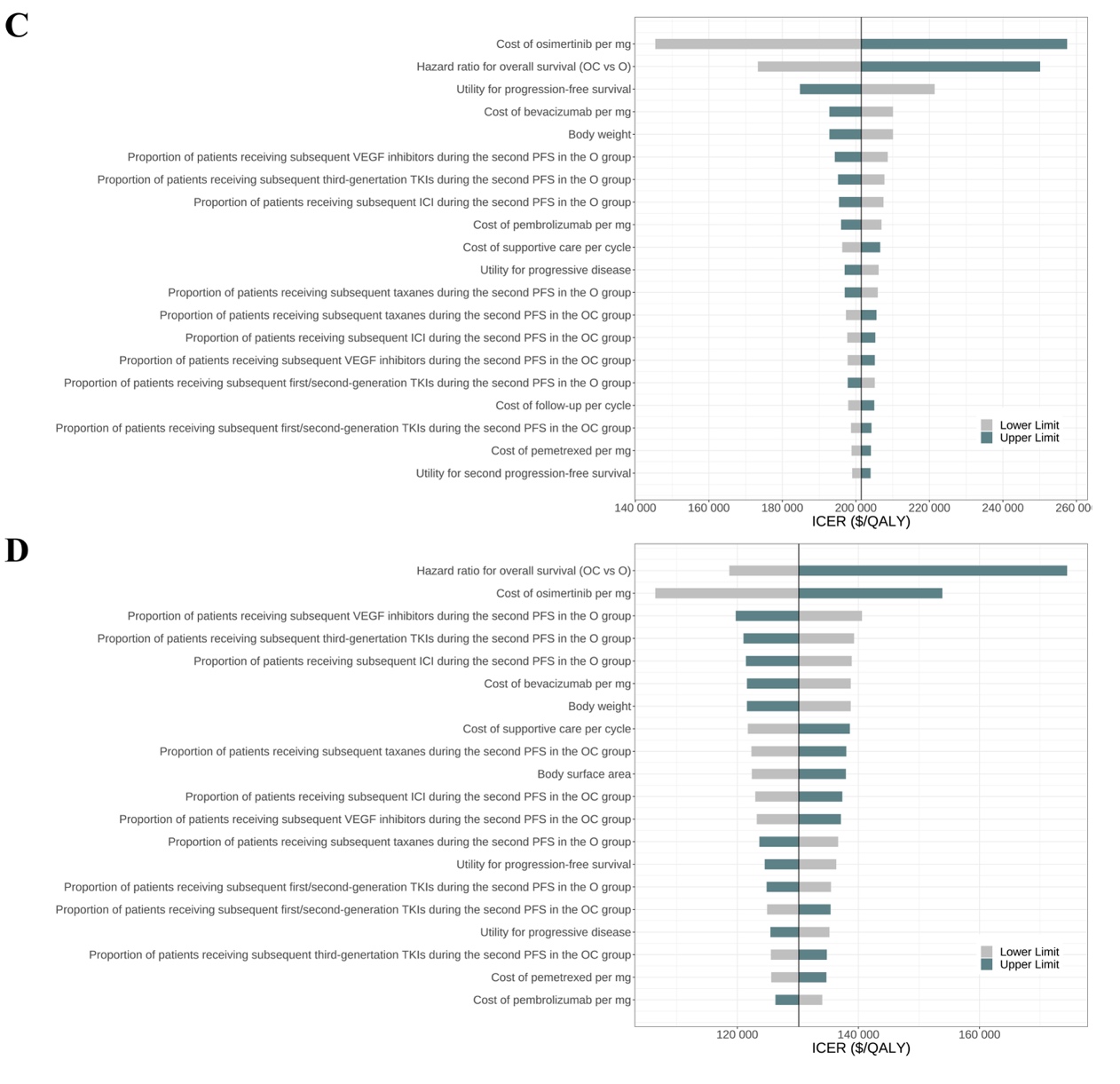


**Figure S6.** One-way Sensitivity Analyses of the Subgroup Analyses. A) Patients with the L858R mutations; B) Patients with Ex19del; C) Patients with CNS metastases; D) Patients without CNS metastases. ***Abbr.*** *ICER, incremental cost-effectiveness ratio; QALY, quality-adjusted life-year; TKI, tyrosine kinase inhibitor; OC, osimertinib plus chemotherapy; O, osimertinib monotherapy; PFS, progression-free survival.*


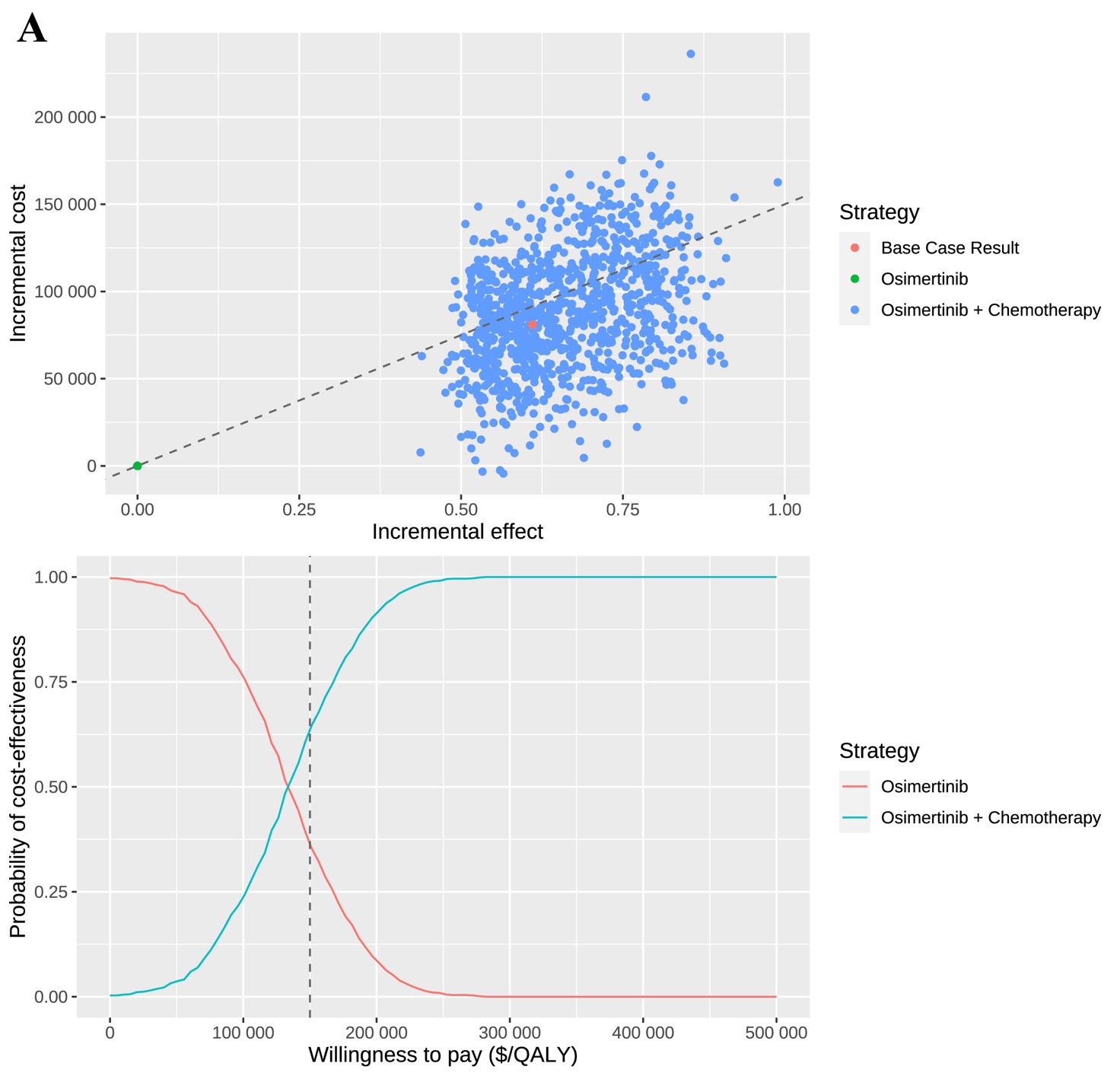

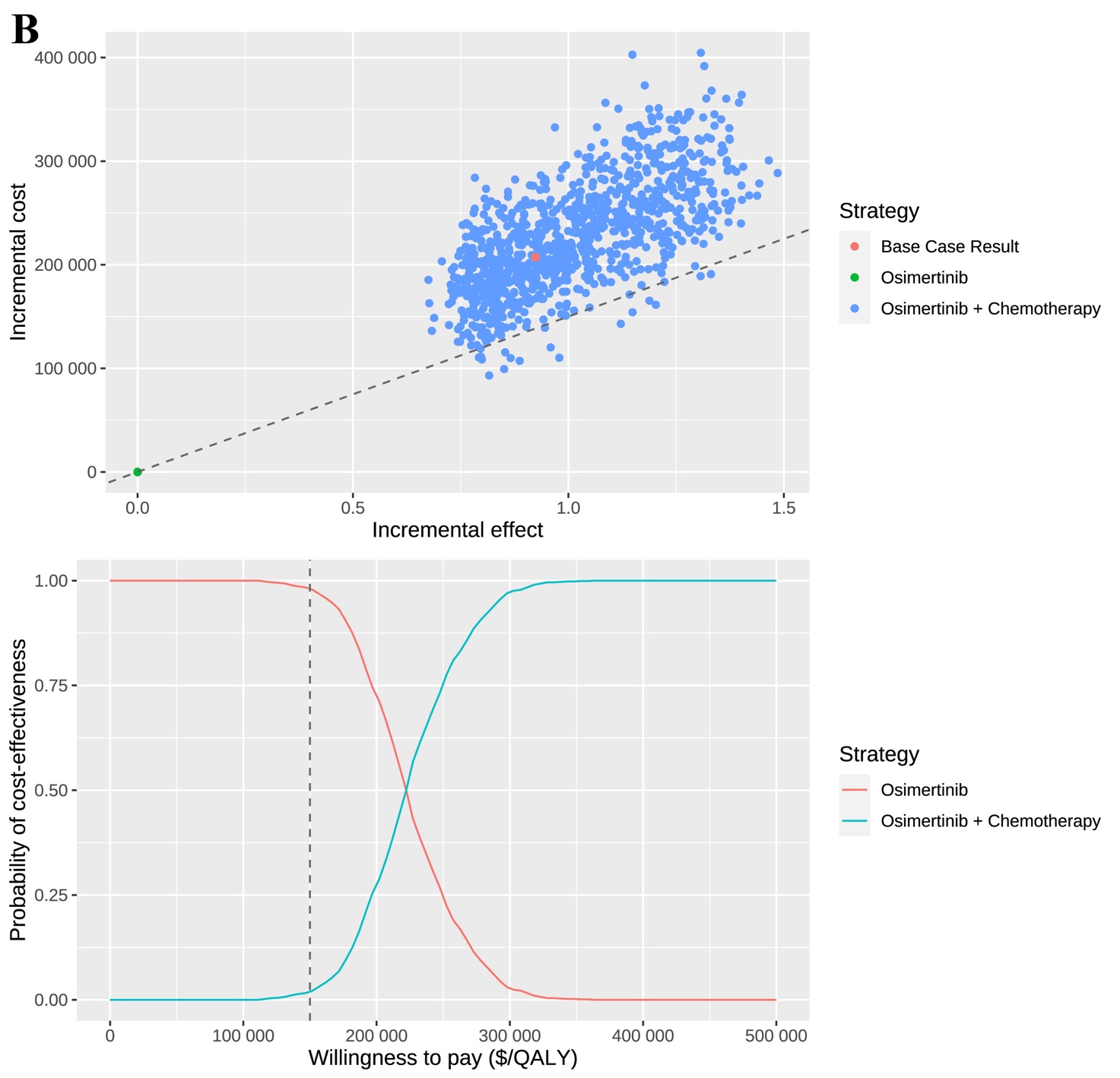

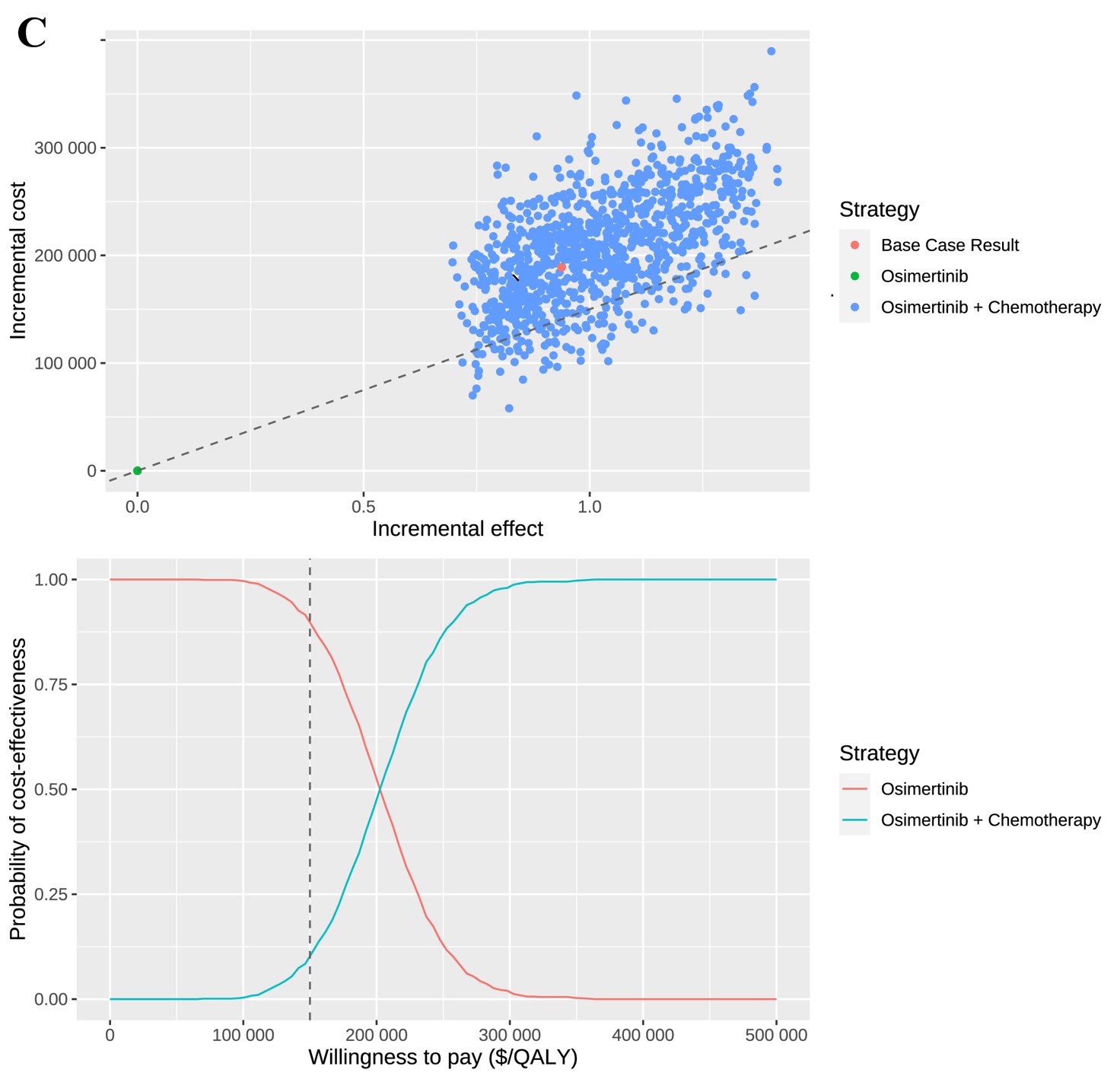


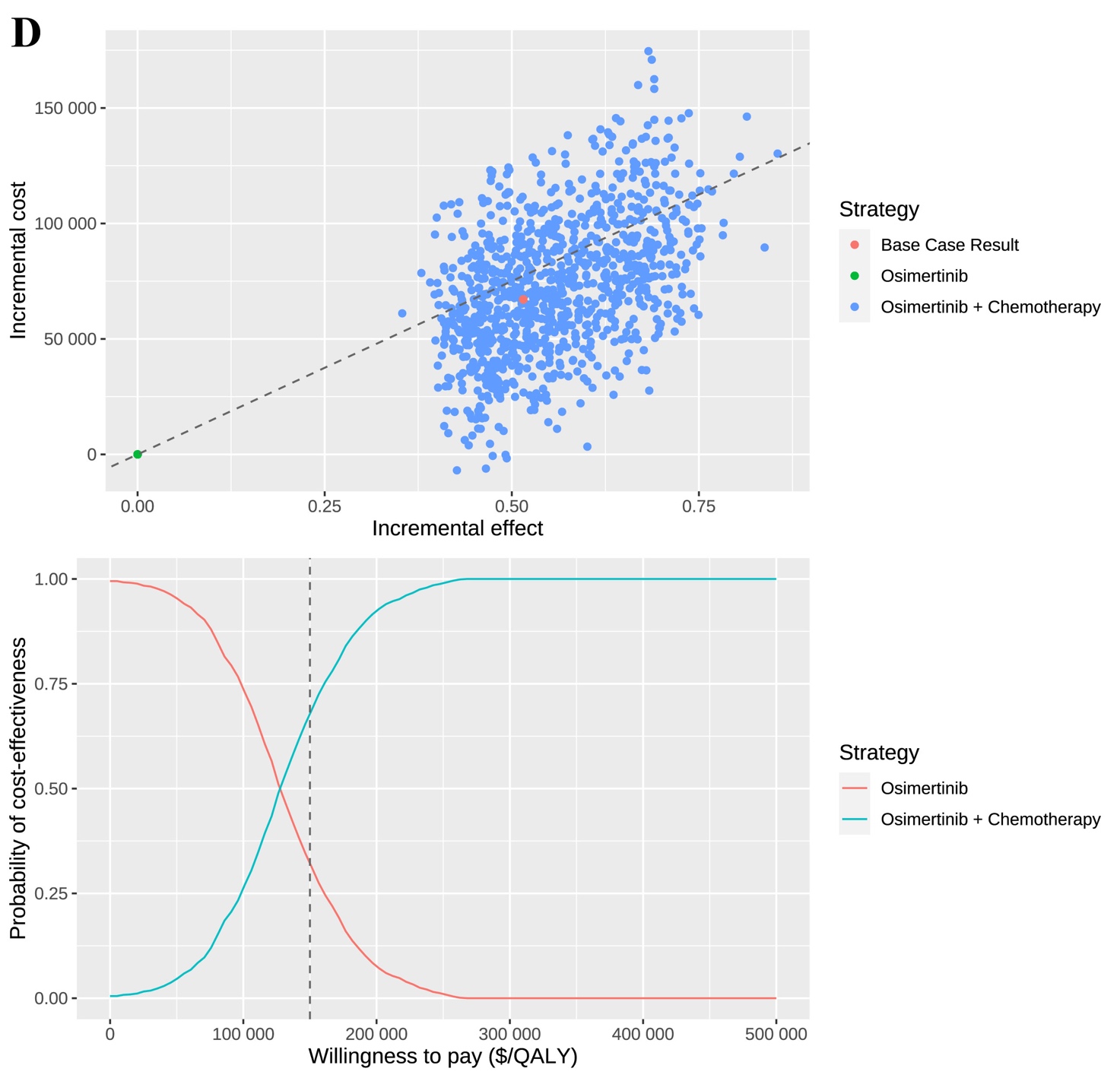


**Figure S7.** Probabilistic Sensitivity Analyses in the subgroup analyses. A) Patients with the L858R mutations; B) Patients with Ex19del; C) Patients with CNS metastases; D) Patients without CNS metastases. The dashed line represents the willingness-to-pay threshold of $150,000 per QALY gained. ***Abbr.*** *QALY, quality-adjusted life-year.*
